# Digenic Variants in the FGF21 Signaling Pathway Associated with Severe Insulin Resistance and Pseudoacromegaly

**DOI:** 10.1210/jendso/bvaa138

**Published:** 2020-09-22

**Authors:** Stephen I Stone, Daniel J Wegner, Jennifer A Wambach, F Sessions Cole, Fumihiko Urano, David M Ornitz

**Affiliations:** 1 Department of Pediatrics, Division of Pediatric Endocrinology & Diabetes, Washington University School of Medicine, St. Louis, Missouri, US; 2 Department of Developmental Biology, Washington University School of Medicine, St. Louis, Missouri, US; 3 Department of Pediatrics, Division of Newborn Medicine, Washington University School of Medicine, St. Louis, Missouri, US; 4 Department of Medicine, Division of Endocrinology, Metabolism, and Lipid Research, Washington University School of Medicine, St. Louis, Missouri, US; 5 Department of Pathology and Immunology, Washington University School of Medicine, St. Louis, Missouri, US

**Keywords:** FGF21, insulin resistance, pseudoacromegaly, genetics, and fibroblast growth factors

## Abstract

Insulin-mediated pseudoacromegaly (IMPA) is a rare disease of unknown etiology. Here we report a 12-year-old female with acanthosis nigricans, hirsutism, and acromegalic features characteristic of IMPA. The subject was noted to have normal growth hormone secretion, with extremely elevated insulin levels. Studies were undertaken to determine a potential genetic etiology for IMPA. The proband and her family members underwent whole exome sequencing. Functional studies were undertaken to validate the pathogenicity of candidate variant alleles. Whole exome sequencing identified monoallelic, predicted deleterious variants in genes that mediate fibroblast growth factor 21 (FGF21) signaling, *FGFR1* and *KLB,* which were inherited in trans from each parent. FGF21 has multiple metabolic functions but no known role in human insulin resistance syndromes. Analysis of the function of the *FGFR1* and *KLB* variants in vitro showed greatly attenuated ERK phosphorylation in response to FGF21, but not FGF2, suggesting that these variants act synergistically to inhibit endocrine FGF21 signaling but not canonical FGF2 signaling. Therefore, digenic variants in *FGFR1* and *KLB* provide a potential explanation for the subject’s severe insulin resistance and may represent a novel category of insulin resistance syndromes related to FGF21.

Extreme insulin resistance syndromes represent a diverse category of diseases. Studying the genetics and molecular biology of such patients has enhanced our collective understanding of the mechanisms of insulin signaling. The best-known examples of extreme insulin resistance syndromes are mediated at the level of the insulin receptor (INSR). These conditions, Donohue syndrome (leprechaunism) and Rabson-Mendenhall syndrome, include growth impairment and short stature and illustrate the important roles of insulin in both linear growth and regulating blood glucose [1, 2]. Unlike INSR-mediated insulin resistance syndromes, a rare subset of patients with insulin-mediated pseudoacromegaly (IMPA) first described by Flier and colleagues in 1993 [3] exhibit tall stature, physical signs of growth hormone excess (acromegaly), and normal growth hormone secretion [3-9]. To date, the molecular mechanism for IMPA remains unknown. However, it has long been hypothesized that these patients must harbor a defect downstream of the INSR in which the glucose-lowering property of insulin is impaired, while the growth-promoting activity of insulin is preserved [10].

Here, we describe a 12-year-old female with IMPA. Whole exome sequencing of the proband, parents, and full female sibling revealed that the proband and her sister carry rare and predicted deleterious variants in the *FGFR1* and *KLB* genes. Notably, FGFR1 and KLB form the receptor complex for fibroblast growth factor 21 (FGF21). FGF21 is a member of the endocrine fibroblast growth factor subfamily [11]. FGF21, a hormone primarily secreted by the liver, acts to regulate the body’s response to starvation through the central nervous system (CNS) and peripheral target tissues [12-14]. FGF21 promotes insulin sensitivity in muscle and adipose tissue by promoting glucose uptake and fatty acid oxidation [12-14]. FGF21 signaling in the CNS results in weight loss and reduced circulating glucose and insulin concentrations [15]. FGF21 also contributes to growth hormone resistance via downregulation of hepatic STAT5 and insulin-like growth factor 1 (IGF1) and upregulation of insulin-like growth factor binding protein 1 (IGFBP1) [15]. Therefore, resistance to FGF21 may explain the proband’s severe insulin resistance and tall stature. Recently, studies have suggested that obesity is an FGF21 resistant state, suggesting that FGF21 may play a role in common forms of insulin resistance [16].

Here, we characterize the metabolic abnormalities of the proband, her parents, and her sister and perform in vitro functional studies of the predicted deleterious *FGFR1* and *KLB* variants. We found that the proband has elevated serum FGF21 and that cells co-transfected with her *FGFR1* and *KLB* variants had an attenuated response to FGF21. In aggregate, these results are the first to demonstrate a potential genetic cause for IMPA and extend our understanding of the important role FGF21 plays in human insulin signaling. Understanding the mechanisms by which FGF21 contributes to insulin sensitivity in humans will facilitate discovery of novel pharmacologic strategies to activate the FGF21 signaling pathway and thereby potentiate insulin sensitivity and secretion.

## 1. Materials and Methods

### A. Institutional Oversight

The studies performed in this report were approved by the Human Research Protection Office at Washington University. We obtained informed written consent from the parents and assent from the siblings for participation in these studies and sharing of photographs.

### B. Clinical Biochemical Testing

All clinical laboratory tests were performed in the clinical laboratory at St. Louis Children’s Hospital.

### C. Oral Glucose Tolerance Testing

The proband completed an overnight fast and baseline serum glucose, insulin, and growth hormone levels were obtained. The proband was instructed to consume 75 g of oral glucose tolerance solution within 5 minutes. Serum glucose, insulin, and growth hormone levels were sampled every 30 minutes for the next 120 minutes. The proband, her parents, and her sister also underwent testing for glucose and insulin 30 to 60 minutes after consuming a high-carbohydrate meal.

### D. Exome Sequencing

Genomic DNA was isolated from saliva samples obtained from the proband, her parents, and full female sibling. Exome capture was performed using the Nimblegen VCRome v2.1 Exome kit (Roche, Madison, WI) with paired-end sequencing (2 × 125 bp) on an Illumina HiSeq 2500 instrument (Illumina, San Diego, CA). Sequence reads were aligned to the human reference genome sequence (GRCh37/hg19) with 90% of the exome having at least 20× coverage. Variants were annotated with Annovar (http://annovar.openbioinformatics.org/en/latest/) [17].Variants in coding regions and near exon-intron junctions that were novel or rare (minor allele frequency less than 0.01 in the Exome Aggregation Consortium [ExAC]) [18] database (exac.broadinstitute.org) or Genome Aggregation Database (gnomAD) (https://gnomad.broadinstitute.org) [19] were assessed for predicted pathogenicity using Combined Annotation Dependent Depletion (CADD, cadd.gs.washington.edu) [20], SIFT (sift.jcvi.org) [21], Polyphen2 (genetics.bwh.harvard.edu/pph2/) [22], LRT (genetics.wustl.edu/jflab/lrt_query.html) [23], MutationTaster (www.mutationtaster.org) [24], GERP++ (mendel.stanford.edu/SidowLab/downloads/gerp/) [25], and PhyloP (http://compgen.cshl.edu/phast/help-pages/phyloP.txt) [26]. Exonic variants were classified as deleterious if predicted to be pathogenic by the majority of these *in silico* programs. Candidate variants were verified by Sanger sequencing.

### E. Protein Sequence Conservation

RefSeq files for orthologs of *FGFR1* and *KLB* were downloaded from NCBI (https://www.ncbi.nlm.nih.gov/search/). MegAlign Pro (DNASTAR, Madison, WI) software was used to align the sequences and generate figures.

### F. FGF21 Enzyme-Linked Immunosorbent Assay

Fasting serum samples were drawn from the proband and her parents. Serum FGF21 levels were determined using the Human Fibroblast Growth Factor 21 enzyme-linked immunosorbent assay (ELISA) (#RD191108200R, BioVendor, Brno, Czech Republic). Samples were prepared in duplicate.

### G. Smell Identification Testing

All 4 subjects underwent smell testing using the University of Pennsylvania Smell Identification Test (UPSIT) [27].

### H. Expression Vectors

Bicistronic expression plasmids were created with the assistance of Bio Basic Inc. (Markham, ON, Canada). The plasmids were designed to express human cDNA for *FGFR1c* (NM_015850) and *KLB* (NM_175737). Gene expression was driven under the CMV promoter. A shortest porcine teschovirus-1 2A-glycine-serine-glyceine (P2A-GSG) linker sequence was added between the *FGFR1* and *KLB* sequences. The P2A-GSG is a self-cleaving linker peptide that allows for equimolar expression of 2 genes under the same promoter [28]. A Flag tag was added to the carboxy-terminus of *KLB.* The *FGFR1* c.304G>A and *KLB* c.26C>A variants identified in the proband were generated alone and in combination via site-directed mutagenesis. From here forward, these plasmids will be abbreviated *FGFR1-P2A-KLB*. Sequencing files with these vectors are available in the supplementary data [29]. The vector backbone was pcDNA3.1(+). The pmaxGFP Vector control (Lonza, Basel, Switzerland) was used as a transfection and negative control.

### I. Cell Lines

Wild-type L6 myoblasts (CRL-1458, rat skeletal muscle myoblasts) and HeLa (CCL-2, human cervical adenocarcinoma cells) were obtained from American Type Culture Collection (ATCC, Manassas, VA). The cells were cultured in DMEM (#30–2002, ATCC) with 10% Heat Inactivated Fetal Bovine Serum (HI FBS, #10082147, Thermo Fisher Scientific) and 1% Penicillin/Streptomycin (#15140163, Thermo Fisher Scientific).

### J. Transient Transfection

Untransfected cells were seeded at a density of 8 × 10^4^ cells/mL in 100 µL of DMEM with 10% HI FBS and cultured in 96-well tissue culture plates (TPP Z707910, Millipore Sigma, Burlington, MA). After overnight recovery, L6 cells were transfected with L6 Cell Avalanche Transfection Reagent (EZ Biosystems, College Park, MD). Transfection complexes were formed according to manufacturer’s instructions using 0.01 µg/µL plasmid DNA and 0.016 µL (reagent)/µL Opti-MEM (#31985088, Thermo Fisher Scientific). After overnight recovery, HeLa cells were transfected with *Trans*IT-X2 Transfection Reagent (MIR6000, Mirus Bio, Madison, WI). Transfection complexes were formed according to manufacturer’s instructions using 0.01 µg/µL plasmid DNA and 0.02 µL (reagent)/µL Opti-MEM. For both cell lines, 10 µL transfection complex was added to each well. After incubating at 37 °C for 5 hours, the media containing the transfection complex was aspirated off and replaced with DMEM with 10% HI FBS.

### K. Phosphorylated-ERK ELISA

Thirty-six hours post transient transfection the L6 myoblasts were serum starved overnight in Opti-MEM. The cells were treated with 100 nM recombinant human FGF21 (#100–42, Peprotech, Rocky Hill, NJ) or 5000 pM recombinant human FGF2 (#100-18B, Peprotech). Experiments testing FGF2 contained Opti-MEM and 2µg/mL heparin (#H3149, MilliporeSigma) in both the vehicle and FGF2 conditions. Cells were treated with FGF2 or FGF21 for 10 minutes. Subsequently, the media was aspirated and removed. Phosphorylated-ERK (pERK)1/2 was measured using AlphaLISA SureFire Ultra p-ERK 1/2 High Volume kit (ALSU-PERK-A-HV, PerkinElmer, Waltham, MA) according to the manufacturer’s instructions. The cells were lysed in 70 µL of lysis buffer. Then 30 µL of lysate were transferred to a 96-well 1/2 area plate (#6005760, PerkinElmer, Waltham, MA) for subsequent analysis. Plates were read on a BioTek Synergy 2 microplate reader (Winooski, VT) with an excitation at 680 nm and emission at 620 nm. pERK results were normalized to green fluorescent protein (GFP)-expressing cells treated with vehicle alone.

### L. Statistics

Except where otherwise noted, *P* ≤ 0.05 was considered significant*. P* values were calculated using Prism 8 Software (GraphPad, La Jolla, CA) using a 2-way analysis of variance (ANOVA) with multiple comparisons performed via a Tukey Honest Significant Difference test. *P* values in figures demonstrate the following abbreviations: *ns* > 0.05, * ≤ 0.05, ** ≤ 0.01, *** ≤ 0.001, **** ≤ 0.0001.

## 2. Results

### A. Case Presentation

**Figure 1.**
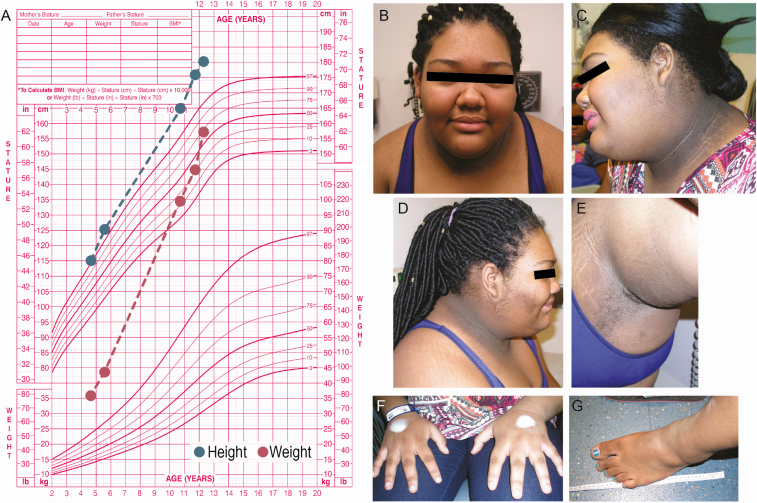
**Clinical features of the proband with IMPA. A)** Growth chart constructed from available growth points (based on Centers for Disease Control Girls 2-20 years chart). Height is plotted in blue, while weight is plotted in red. Patients photographs taken from 2 separate clinic visits. **B)** Facial features demonstrate widely spaced eyes and frontal bossing. Left profile **(C)** and right profile **(D)** demonstrate severe acanthosis nigricans, facial hirsutism, and acne. **E)** Left axillae with acanthosis nigricans. Large hands* **(F)** and feet **(G)**. *The white color on the dorsum of the proband’s hands is EMLA cream in preparation for IV placement.

The proband was born at full term with a normal birth weight of 6 pounds (lb) 7 ounces (2920 g; 15th percentile). She was the product of a nonconsanguineous relationship between a Hispanic woman and an African American man. The proband was reportedly larger than her peers since age 5. Around 10 years of age, at the time of adrenarche, the proband began rapidly to gain weight. Simultaneously, she underwent a rapid height acceleration (Figure 1A). At 12 years of age, the proband’s primary care provider noted she had an elevated random blood glucose and was hypertensive. She was started on metformin and lisinopril and referred to our pediatric endocrinology clinic at St. Louis Children’s Hospital.

Upon evaluation the patient was noted to have extremely large stature for her age. Her height was 185 cm (72 inches, Z = +4.26), weight was 128.2 kg (282.6 lb, Z = +3.46), and body mass index (BMI) 37.46kg/m^2^ (Z = +2.55) (Table 1). The proband exhibited many striking features, including prominent frontal bossing with coarsened facial features, widely spaced eyes, and hirsutism including moderate facial acne and facial hair present on her sideburns, cheeks, upper lip, chin, and neck. Severe acanthosis nigricans was present on her neck, nasal ridge, axillae, groin, and chest. Her hands were widened with large fingers. She had large feet, wearing men’s US size 13 shoes (Figure 1B-G). Notably, she had central adiposity with relative sparing of obesity from her lower extremities. However, there were no areas devoid of subcutaneous fat (Supplementary Figure 1A-1C [29]).

**Table 1.** Kindred With Insulin-Mediated Pseudoacromegaly: Age, Anthropometric Measurements, Insulin, and Glucose Levels in This Kindred

	Mother	Father	Sibling	Proband
Age (years)	27	31	8	12
Height (cm) (Z-score)	165.1 (+0.30)	182 (+0.94)	143.4 (+2.0)	181.5 (+3.34)
Weight (kg) (Z-score)	104.3 (+2.30)	99.8 (+1.99)	58.8 (+7.62)	132.0 (+11.03)
BMI (kg/m^2^) (Z-score)	38.3 (+2.19)	29.8 (+1.77)	28.6 (+3.91)	39.3 (+4.19)
Insulin (μIU/mL)	285	99.2	414.9-438	1279-2446
Glucose (mg/dL)	102	110	117	255

Abbreviation: BMI, body mass index.

The proband’s father is an African American man with history of hypertension. Her mother is a Caucasian woman of Hispanic heritage with a history of obesity and irregular menses. She recently underwent bariatric weight loss surgery. Pictures of the proband and each parent are shown in Supplementary Figure 1D-1E [29]. The proband’s sister also has a similarly large stature for her age and is starting to show early signs of weight gain and acanthosis nigricans at age 8 years.

### B. Clinical Workup

Due to the proband’s extreme growth pattern, we hypothesized that she had a syndrome of growth hormone excess (acromegaly). However, her IGF1 and insulin-like growth factor binding protein 3 (IGFBP3) were both normal at 331 ng/mL and 5.3 μg/mL respectively (Table 2). While continuing treatment with 1000 mg metformin twice daily, the proband underwent a growth hormone suppression test (oral glucose tolerance test) (Table 3). Her serum growth hormone became undetectable at 90 minutes, effectively ruling out the possibility of growth hormone excess. A bone age radiograph was interpreted as 14 years, demonstrating near closure of the epiphyses (Supplementary Figure 2 [29]).

**Table 2.** Biochemical Laboratory Testing: Relevant Laboratory Values of the Proband

Laboratory Test	Value	Normal Range
HbA1c (%)	6.6	4.0-5.7
Glucose (mg/dL)	186	70-105^b^
IGF1 (ng/mL)	331	178-636
IGFBP3 (μg/mL)	5.3	2.6-8.6^a^
Insulin (μIU/mL)	1279-2446	2.0-19.6^b^
DHEA-S (μg/dL)	99	45-320^a^
Total testosterone (ng/dL)	66	<33^a^
Free testosterone (pg/mL)	16	0.1-7.4
17-hydroxyprogesterone (ng/dL)	69	<169
Estradiol (pg/mL)	29	34-170^a^
LH (μIU/mL)	7.7	0.4-11.7^a^
FSH (μIU/mL)	5.4	1.0-9.2^a^
Total cholesterol (mg/dL)	141	120-170
Triglycerides (mg/dL)	189	0-150^b^
HDL (mg/dL)	29	35-60
LDL (mg/dL)	74	1-130
AST (U/L)	31-59	12-45
ALT (U/L)	72-126	0-55
Leptin (ng/mL)	19	3.3-18.3
Adiponectin (μg/mL)	2	4-22

Abbreviations: ALT, alanine aminotransferase; AST, aspartate aminotransferase; DHEA-S, dehydroepiandrosterone sulfate; FSH, follicle-stimulating hormone; HbA1c, glycated hemoglobin A1c; HDL, high-density lipoprotein; IGF1, insulin-like growth factor 1; IGFBP3, insulin-like growth factor binding protein 3; LDL, low-density lipoprotein; LH, luteinizing hormone.

^a^Tanner Stage 5 Range

^b^Fasting normal range

**Table 3.** Oral Glucose Tolerance/Growth Hormone Suppression Testing

Time (minutes)	0	30	60	90	120
Glucose (mg/dL)	85	142	127	128	120
Insulin (mIU/mL)	27.7^a^	752^a^	799	488	390
Growth Hormone (ng/mL)	0.55	0.24	0.1	<0.1	0.49

Glucose, insulin, and growth hormone were measured at baseline and every 30 minutes over 2-hour period after ingestion of a 75 g oral glucose.

^a^The 0- and 30-minute samples were hemolyzed, which may spuriously lower the insulin levels.

During the growth hormone suppression test, the proband’s fasting insulin level was only mildly elevated at 27.7 μIU/mL. However, the postprandial samples were extremely elevated (752, 799, 488, and 390 μIU/mL at 30, 60, 90, and 120 minutes, respectively) (Table 3). Notably, there was hemolysis at the 0 and 30-minute time points, which may have spuriously lowered insulin levels in these samples [30]. On multiple occasions we have measured postprandial insulin levels on the proband. We consistently have measured grossly elevated postprandial insulin levels ranging between 1279 and 2446 μIU/mL (Table 2).

Despite the proband’s apparently severe insulin resistance, she has relatively mild diabetes. At diagnosis, her hemoglobin A1c was 6.6%. During the oral glucose tolerance test, her serum glucose peaked at 142 mg/dL (Table 2). On review of home blood glucose records, the proband’s blood glucose measures were usually in the mid 100 mg/dL range, but intermittently spiked into the 300 to 400 mg/dL range after consuming concentrated carbohydrates.

Due to the proband’s signs of hirsutism, we measured dehydroepiandrosterone sulfate (DHEA-S) level, which was normal at 99 μg/dL. The total testosterone was elevated at 66 ng/dL, and the free testosterone was also elevated at 16 pg/mL. Her 17-hydroxyprogesterone level was 69 ng/dL, ruling out late-onset congenital adrenal hyperplasia. The proband’s luteinizing hormone (LH) level was elevated compared to her follicle-stimulating hormone (FSH) level (7.7 μIU/mL and 5.4 μIU/mL, respectively). Her estradiol was normal for age (29 pg/mL) (Table 2). Clinically, the proband had developed to Tanner stage V breast and pubic hair development. Despite reaching full physical maturity, the proband had yet to experience her first period. The proband experienced menarche after completing a medroxyprogesterone withdrawal. Regular menstrual cycles were triggered after treatment with Ortho-Cyclen and spironolactone.

Due to the proband’s clinical features of metabolic syndrome, we obtained a fasting lipid panel. Her total cholesterol was 141 mg/dL, triglycerides were elevated at 189 mg/dL, and high-density lipoprotein (HDL) was low at 29 mg/dL. Her low-density lipoprotein (LDL) was 74 mg/dL (Table 2).

Due to her insulin resistance, we also evaluated the proband for signs of nonalcoholic fatty liver disease. The proband initially had a normal aspartate amino transferase (AST) level (31 U/L) and a mildly elevated alanine amino transferase (ALT) level (72 U/L). Gamma glutamyl transferase (GGT) was normal at 27 U/L. A liver ultrasound demonstrated diffusely increased echogenicity consistent with hepatic steatosis. Doppler examination was normal with a main hepatic artery restrictive index of 0.6. Over time, the proband’s hepatic steatosis has worsened with AST increasing to 59 U/L and ALT increasing to 126 U/L. (Table 2). Hepatic synthetic function remains normal with normal prothrombin time and international normalized ratio.

Due to the proband’s lack of lower extremity adiposity and severe insulin resistance, we assayed adipose derived hormones. The proband’s fasting leptin level was 19.0 ng/mL, which is mildly elevated for age, but appears to be below expectations for her degree of obesity [31]. Conversely, her adiponectin level was 2 μg/mL, which is low relative to BMI [32] (Table 2).

The proband was found to have refractory hypertension and began treatment with lisinopril and amlodipine. Her electrocardiogram and echocardiogram were normal. Due to her history of loud snoring, the proband underwent a polysomnogram and was found to have obstructive sleep apnea and was started on bilevel positive airway pressure (BiPAP).

In addition to her medical issues, the proband experienced depression. She was subject to ridicule from her peers. Specifically, she found it difficult to fit into her desk at school and was teased about her body odor. Developmentally, the proband received fair grades. She enjoys participating in her school’s volleyball and softball teams. Unfortunately, she frequently experienced musculoskeletal complaints. Despite traditional efforts at weight loss, the proband was unable to lose any significant weight. The proband often complained of increased appetite and often snacked in between meals.

### C. Genetic Testing

Due to her extreme phenotype and suspicion for an underlying genetic etiology, a chromosomal microarray was obtained and was nondiagnostic. Research whole exome sequencing, performed for the proband, her parents, and her sister, did not identify any de novo or autosomal recessive monogenic variants that accounted for her phenotype. We hypothesized that an oligogenic model of inheritance may account for her phenotype and analyzed whole exome sequencing data for coding variants shared by the proband, her sister, and parents. We identified 1108 predicted deleterious variants shared between the proband, her sister, and one of her parents. This list was narrowed down to 11 candidate variants based on gene set enrichment analysis of relevant insulin and IGF1 pathways (Supplementary Table 1 [29]) [33]. From this list, we selected 2 rare, predicted deleterious variants in genes critical to the FGF21 signaling pathway in *FGFR1* and *KLB*.

**Figure 2.**
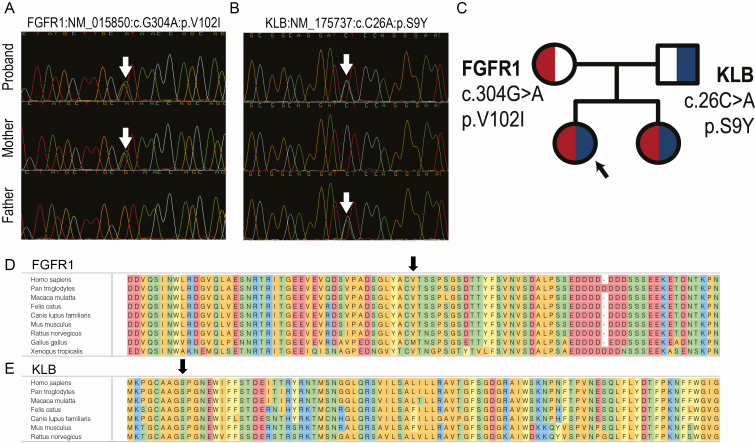
**Genetic analysis of *FGFR1* and *KLB* variants. A)** Sanger tracing demonstrating *FGFR1* c.304G>A (g.39099G>A). **B)** Sanger tracing demonstrating *KLB* c.26C>A (g.123C>A). **C)** Pedigree demonstrating that the proband (arrow) and her sister inherited the *FGFR1* and *KLB* variants in trans from each parent. The missense variants demonstrate corresponding nucleotide and amino acid substitution. **D)** The FGFR1 Valine at the affected position is highly conserved down to *X. tropicalis*. **E)** The KLB Serine at the affected position is highly conserved down to *M. musculus* and *R. norvegicus*.

*FGFR1* and *KLB* form a transmembrane receptor-cofactor complex that binds FGF21 and activates intrinsic tyrosine kinase activity and subsequent signal transduction [34]. The *FGFR1* variant (p.V102I) was maternally inherited, while the *KLB* variant (p.S9Y) was paternally inherited. The proband and her younger sister are heterozygous for variants in both *FGFR1* and *KLB,* inheriting one variant from each parent (trans) (Figure 2A-2C). As noted above, the younger sister is exhibiting early signs of a phenotype similar to the proband. Both of these variants are rare (present in fewer than 2/1000 to 2/10 000 individuals) and are predicted to be damaging by the majority of *in silico* variant prediction programs (Supplementary Table 1 [29]). These variants reside in functional, highly conserved regions of the proteins (Figure 2D-E). As the *FGFR1* and *KLB* variants are inherited in trans from each parent (Figure 2C), we hypothesized that a digenic inheritance model accounted for the phenotype with the variants in *FGFR1* and *KLB* acting synergistically and leading to FGF21 resistance, thereby explaining the proband’s severe insulin resistance and tall stature.

### D. Physical and Biochemical Testing

**Figure 3.**
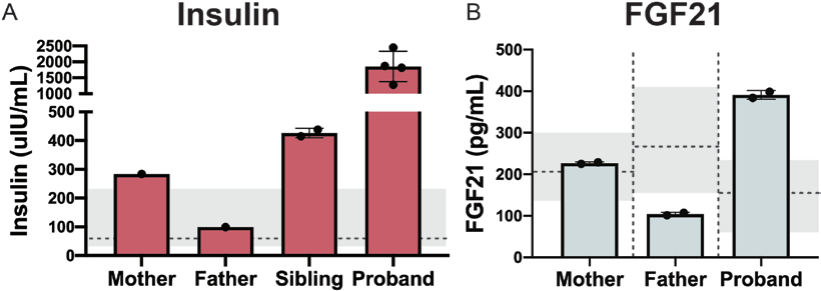
**Biochemical analysis of insulin and FGF21. A)** Postprandial insulin levels obtained 30 to 60 minutes after a high-carbohydrate meal. Normal range is shaded gray. Median value is noted by the dashed line. **B)** Fasting serum FGF21 levels. Age- and sex-specific normal ranges are shaded gray. Median values are noted by the dashed lines.

Anthropomorphic data were obtained from the proband, her sister, and her parents (Table 1). Postprandial serum insulin levels were obtained 30 to 60 minutes after consuming a high-carbohydrate meal. As described above, the proband’s mother (carrying the *FGFR1* variant) had mildly elevated insulin (285 μIU/mL), while the proband’s father (carrying the *KLB* variant) had a normal insulin level (99.2 μIU/mL). As expected, the proband’s insulin was extremely elevated (1279-2446 μIU/mL). Additionally, the sister’s postprandial insulin was elevated (414.9-438μIU/mL), fitting the hypothesis that she has early signs of the severe insulin resistance phenotype [35] (Figure 3A).

To evaluate for potential FGF21 resistance, fasting serum FGF21 levels were obtained and measured by ELISA. There are few studies measuring normal FGF21 levels in children. However, serum FGF21 levels tend to rise with age [36]. The mother’s FGF21 level was normal for age (227.0 pg/mL), while the father’s FGF21 level was slightly low for age (104.3 pg/mL). The proband’s FGF21 was elevated for her age (391.3 pg/mL) [36, 37] (Figure 3B). Unfortunately, the sister was unable to provide a fasting blood sample.

As anosmia has been associated with pathogenic variants in *FGFR1* and *KLB* [38, 39], the subjects underwent smell identification testing. Each family member scored in the normosmic range for their age (Supplementary Figure 3 [29]).

### E. In Vitro Testing of FGFR1 and KLB Variants

**Figure 4.**
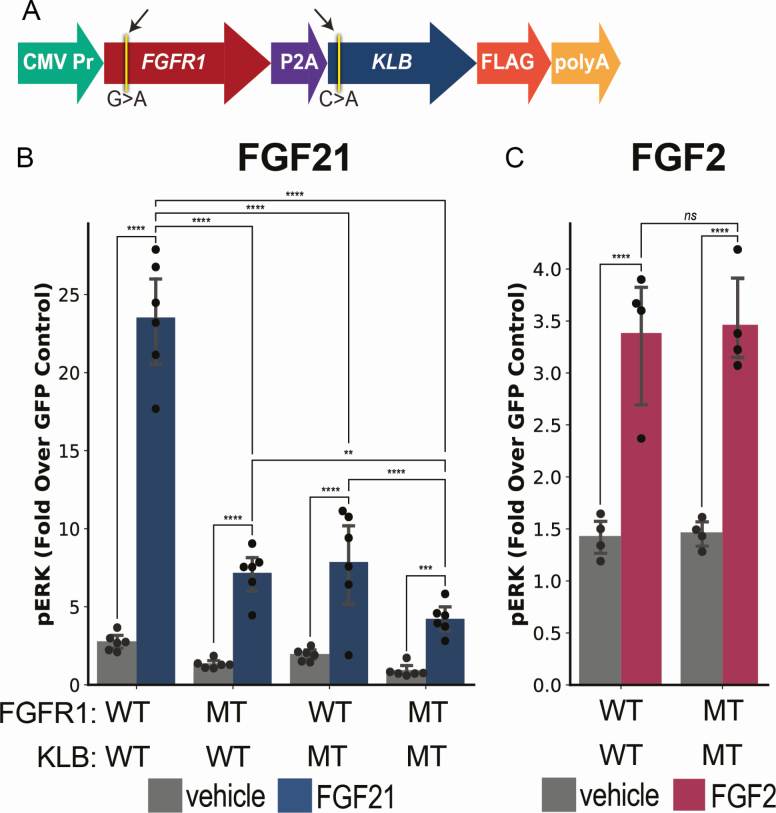
**In vitro analysis of *FGFR1* and *KLB* variants. A)** Schematic of the FGFR1-P2A-KLB plasmid used in the study. L6 myoblasts were transfected with either wild-type (WT) or mutant (MT) FGFR1 / KLB alone and in combination. A GFP expressing plasmid was used as a negative control. **B)** Transfected cells were treated with 100 nM recombinant human FGF21 for 10 min. PhosphoERK (pERK) was measured via ELISA. The results are shown as fold over GFP control. **C)** WT or MT transfected cells were treated with 5000 pM FGF2 for 10 min. pERK was measured via ELISA.

To assess the potential pathogenicity of the FGFR1 and KLB mutations, we designed a bicistronic expression vector (Figure 4A). The plasmids express human cDNA for *FGFR1c* (NM_015850) and *KLB* (NM_175737). The *FGFR1* c.304G>A and *KLB* c.26C>A variants identified in the proband were generated alone and in combination via site-directed mutagenesis (Figure 4A). These plasmids are abbreviated as FGFR1-P2A-KLB.

L6 myoblasts are a rat myoblast cell line that is useful in endocrine FGF research as they express low endogenous levels of FGF receptors, klotho, and beta-klotho [40]. The FGFR1-P2A-KLB plasmid was transiently transfected into L6 myoblasts. A GFP control plasmid was also transfected for use as a transfection negative control. The phosphorylation of extracellular signal-regulated kinase (pERK) is an early response phosphoprotein in the FGF receptor signaling cascade [11], and as a result, pERK is a useful tool for studying FGF ligands and FGF receptors.

To evaluate if the FGFR1 and KLB mutations have an effect on endocrine FGF signaling, we cultured the cells in Opti-MEM and added recombinant human FGF21 (100 nM). When transfected with wild-type FGFR1 and wild-type KLB there was a 2.7-fold (± 0.6) increase of pERK when left untreated. When treated with FGF21 there was a 23.5-fold (± 3.7) increase of pERK (*P* = 1.0 × 10^-7^). Cells transfected with mutant FGFR1 and wild-type KLB demonstrated a 1.3-fold (± 0.1) increase of pERK when left untreated (*P* = 3.8 × 10^–6^). When treated with FGF21 there was a 7.2-fold (± 0.6) increase in pERK (*P* = 3.7 × 10^–6^). Similarly, cells transfected with wild-type FGFR1 and mutant KLB demonstrated a 1.9-fold (± 0.2) increase in pERK when treated with vehicle alone. When treated with FGF21 there was a 7.9-fold (± 1.4) increase in pERK (*P* = 0.0018). Finally, cells transfected with mutant FGFR1 and mutant KLB demonstrated a 0.9-fold (± 0.4) increase over GFP when left untreated. When treated with FGF21 there was only a 4.2-fold (± 1.0) increase in pERK (*P* = 2.1 × 10^–5^). There was a significant decrease in pERK when comparing the wild-type FGFR1 and wild-type KLB transfected cells versus cells expressing either mutant FGFR1 or KLB (*P* = 1.8 × 10^–6^ and 2.0 × 10^–5^, respectively). There was also a significant decrease in pERK when comparing the wild-type transfected cells with cells expressing mutations in both FGFR1 and KLB (*P* = 2.6 × 10^–7^). The pERK response to FGF21 in cells expressing mutations in both FGFR1 and KLB was also significantly lower than in cells expressing mutations only in FGFR1 (*P* = 0.003) or KLB (*P* = 0.03) (Figure 4B).

To evaluate if the FGFR1 and KLB mutations have any effect on canonical FGF signaling, we again transfected the FGFR1-P2A-KLB plasmids into L6 myoblasts. This time, we cultured the cells in the presence of 2 µg/mL heparin and added 5000 pM recombinant human FGF2. Notably, canonical FGFs require the presence of heparan sulfate proteoglycans to bind the FGF receptor [11]. When left untreated, the wild-type FGFR1 and wild-type KLB transfected cells demonstrated a 1.4-fold (± 0.2) increase in pERK over GFP. When treated with FGF2 there was a 3.4-fold (± 0.7) increase in pERK (*P* = 0.002). Similarly, the mutant FGFR1 and mutant KLB transfected cells demonstrated a 1.5-fold (± 0.2) increase in pERK over GFP in the absence of FGF2. When treated with FGF2, there was a 3.5-fold (± 0.5) increase in pERK (*P* = 0.0002). When comparing FGF2 treated cells transfected with wild-type FGFR1 and wild-type KLB to cells transfected with mutant FGFR1 and mutant KLB, there was no significant differences in pERK (*P* = 0.86) (Figure 4C).

Notably, the kindred studied here is heterozygous for missense mutations in both FGFR1 and KLB. As a result, we repeated the ERK phosphorylation studies in HeLa cells, a cell line known to express endogenous FGFR1 and KLB [41, 42]. We found similar results in HeLa cells compared with L6 myoblasts. When transfected with wild-type FGFR1 and wild-type KLB, the vehicle-treated cells had a 4.8-fold (± 0.5) increase in pERK. After treatment with FGF21, there was an 8.8-fold (± 0.3) increase in pERK (*P* = 2.4 × 10^–5^). There was no significant difference comparing vehicle to FGF21 treatment when evaluating mutant FGFR1 alone, mutant KLB alone, or double mutant FGFR1/KLB transfected cells (*P* = 0.12, 0.48, and 0.57, respectively). However, when comparing FGF21-stimulated cells, there was a significant decrease in pERK when comparing wild-type cells to mutant FGFR1 alone (3.7 ± 0.8), mutant KLB alone (2.9 ± 0.6), or double mutant FGFR1/KLB transfected cells (2.9 ± 0.6) (*P* = 0.0001, 8.1 × 10^–6^, and 4.2 × 10^–9^, respectively) (Supplementary Figure 4 [29]). These findings suggest that the FGFR1 and KLB mutations may function as dominant negatives.

## 3. Discussion

This report describes an adolescent female with severe insulin resistance and clinical features of acromegaly. We have conducted biochemical and genetic studies aimed at defining the genetic and molecular etiology of this disorder.

We identified that the affected proband carries monoallelic, digenic variants in the FGFR1 and KLB genes. *FGFR1* and *KLB* form the receptor complex for FGF21 [11], an important metabolic signaling molecule that regulates the mammalian response to starvation [43]. We found that the affected proband has extremely elevated insulin and elevated serum FGF21 for age. In vitro studies demonstrate that the *FGFR1* and *KLB* variants are hypomorphic leading to reduced FGF21 signaling. These studies suggest that the variants in *FGFR1* and *KLB* induce resistance to the insulin-sensitizing effects of FGF21.

Insulin resistance occurs when a greater than normal amount of insulin is required to elicit a quantitatively normal response. Insulin resistance is a common feature of many chronic metabolic conditions [44]. However, there are several genetic syndromes of severe insulin resistance that have illuminated our understanding of the basic biology of insulin signaling and resistance, for example, the autosomal recessive insulin receptor (INSR) defects seen in Donohue syndrome and Rabson-Mendenhall syndromes. Notably, these conditions illustrate the role of INSR signaling in growth, as linear growth retardation is a common feature of these conditions. Autosomal dominant insulin resistance syndromes include Type A Insulin Resistance [45] and HAIR-AN [46] syndromes. Severe insulin resistance can occur in the setting of severe obesity syndromes resulting from pathogenic variants in *MC4R* [47], *POMC* [48], *LEP* [49], and *LEPR* [50]. Additionally, severe insulin resistance is found in generalized and partial lipodystrophy syndromes associated with pathogenic variants in *AGPAT2* [51], *LMNA* [52], and *PPARG* [53]. There appears to be sexual dimorphism in severe insulin resistance syndromes, as women often experience ovarian dysfunction and hyperandrogenism that lead to an earlier clinical presentation [54]. Pseudoacromegaly has been described in congenital generalized lipodystrophy [55]. Pseudoacromegaly has also been described in patients suffering from Sotos syndrome resulting from pathogenic variants in the *NSD1* gene [56].

Insulin-mediated pseudoacromegaly (IMPA) represents a distinct biochemical and physical phenotype when compared with other insulin resistance syndromes due to extreme postprandial insulin resistance, severe acanthosis nigricans, and hyperandrogenism in the setting of extreme tall stature, obesity, and overgrowth.

FGF21 is a hormone primarily secreted by the liver that acts to regulate the body’s response to starvation through CNS and peripheral target tissues [12-14]. Increased levels of FGF21 are associated with obesity, fatty liver, atherogenic lipid profiles, and reduced bone mineral density. This suggests that metabolic syndrome is an FGF21-resistant state [57, 58]. Pharmacologic administration of FGF21 imparts resistance to high-fat-diet–induced weight gain, improves glucose tolerance and hepatic and peripheral insulin sensitivity (without triggering hypoglycemia), and normalizes hyperinsulinemia and hypertriglyceridemia. Interestingly, resistance to FGF21 is similar to high circulating insulin and leptin concentrations in insulin- and leptin-resistant states, respectively [58].

This is the first report to identify a potential molecular etiology for IMPA. Functional variants in *FGFR1* and *KLB* are a biologically plausible explanation for this condition by linking the postprandial insulin resistance and overgrowth phenotypes. FGF21 increases insulin sensitivity in muscle and adipose tissue by promoting glucose uptake and fatty acid oxidation [59, 60]. FGF21 promotes glucose uptake via a mechanism independent of insulin by stimulating the expression of GLUT1>GLUT4 in white adipose tissue [61]. In contrast, insulin stimulates glucose uptake via GLUT4 expression [62]. In this scenario insulin’s GLUT4 mediated effects remain intact, hence explaining why the proband is not extremely hyperglycemic. This is consistent with the observation that in humans (compared with nonhuman primates and mice) FGF21 does not lower blood glucose levels [63, 64]. FGF21 also contributes to growth hormone resistance via downregulation of hepatic STAT5 and IGF1 and upregulation of IGFBP1 [65]. Therefore, loss of FGF21 signaling could lead to disinhibition of growth hormone signaling and increased linear growth.

FGF21 is thought to regulate PPARγ, which is a regulator of adipogenesis. Interestingly, subjects with familial partial lipodystrophy type 3, a disease linked to pathogenic variants in *PPARG*, have striking clinical similarity to our proband with IMPA [66, 67]. This may explain the lipodystrophy-like phenotype observed in the proband, including her relative lack of lower extremity adiposity and low leptin levels relative to her BMI. FGF21 also signals via PPARα in the liver which may explain the development of nonalcoholic steatohepatitis (NASH) in the proband. Interestingly, FGF19 is another endocrine FGF that is important for the regulation of bile acid synthesis. It acts mainly via FGFR4 and KLB. Therefore, mutant KLB may also be affecting FGF19 to potentiate the NASH phenotype. Recently published data suggest that FGF21 mediates many of its effects via the CNS driving thermogenesis and insulin sensitivity [15]. Therefore, defective CNS FGF21 signaling may explain the proband’s propensity to gain weight and overall lack of satiety.

**Figure 5.**
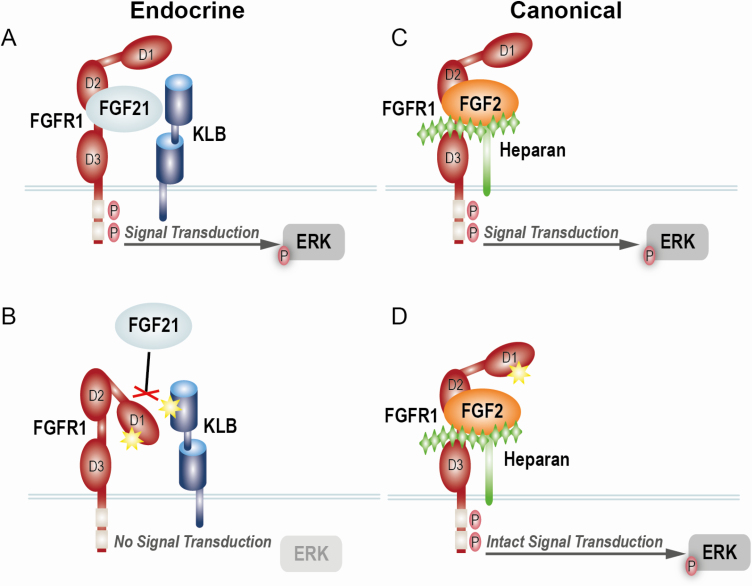
**Model of *FGFR1* and *KLB* effect on endocrine vs canonical FGF signaling. A)** The endocrine FGF (FGF21) requires the binding of FGFR1 and a transmembrane cofactor KLB. This allows for diffusion through the blood stream and mediates its endocrine effects. **B)** The D1 domain of FGFR1 acts to sterically inhibit binding of FGF21 to the binding pocket between the D2 and D3 domains. KLB helps facilitate this interaction, strongly potentiating the binding of FGF21 to FGFR1. Binding of FGF21 to the FGFR1-KLB receptor complex activates an intrinsic tyrosine kinase domain leading the downstream signaling, including the phosphorylation of the extracellular signal-regulated kinase (ERK). Hypothetically the missense mutations may prevent the formation of the FGFR1-KLB receptor complex. **C)** In contrast to endocrine FGF signaling, canonical FGF signaling (i.e., FGF2) depends on heparin/heparan sulfate as a cofactor. Thus, this acts in a paracrine fashion. Binding of FGF2 to the FGFR1-Heparin complex stimulates activation of the tyrosine kinase domain, and subsequent ERK phosphorylation. **D)** Hypothetically heparan sulfate is able to overcome the FGFR1 missense mutation leading to intact downstream signaling.

Here, we present a model for the proposed effects of FGFR1 and KLB mutations on endocrine FGF21 signaling in comparison to canonical FGF2 signaling (Figure 5). In this model, canonical FGF2 signaling remains intact as it relies on heparin/heparan sulfate as a primary cofactor instead of KLB. Therefore, the growth-promoting, paracrine effects of canonical FGFs remain unchecked. It is known that FGF21 stimulates glucose uptake via GLUT1 [61]. Therefore, we hypothesize that mutant FGFR1 and KLB could lead to decreased GLUT1 and decreased FGF21 dependent glucose uptake (FGF21 resistance). This could result in an increased reliance on insulin-mediated glucose uptake, via GLUT4 and subsequent hyperinsulinemia. It is known that supraphysiologic insulin levels can bind nonspecifically to IGF1R and hybrid (insulin/IGF1) receptors preferentially activating growth-promoting pathways [68, 69]. Further studies aimed at testing the effects of these variants on GLUT1/4 expression and translocation to the cell surface are warranted.

Pathogenic variants in *FGFR1* and *KLB* have individually been described in the setting of Kallman syndrome, a disorder characterized by hypogonadotropic hypogonadism and loss of smell (anosmia). These variants, especially in females, demonstrate incomplete penetrance. However, the majority of patients with variants in either *FGFR1* and *KLB* exhibit obesity, dyslipidemia, and insulin resistance [38, 39, 70]. Additionally, there has been one case of Kallman syndrome in which the affected patient carried both a missense variant in *FGFR1* (R78C) and a single amino acid deletion in *KLB* (F777delF). These variants were shown to be hypomorphic both individually and in combination [70].

The FGFR1-KLB receptor complex forms as a heterodimer/tetramer comprising 2 copies of FGFR1 and 2 copies of KLB. As the proband is heterozygous at each allele, we anticipate that her FGFR1-KLB receptor complexes will be an admixture of both wild-type and mutant proteins. As the presence of wild-type FGFR1 and KLB did not rescue ERK phosphorylation in HeLa cells, we postulate that these missense mutations could function as direct or indirect dominant negative receptors. The tissue culture systems that we have created do not fully recapitulate the heterozygous aspect of the disease. Therefore, future studies aimed at generating an animal expressing the *FGFR1* and *KLB* variants will help to answer this question.

The FGFR1 mutation is located in the D1 extracellular domain of the protein. The D1 domain is thought to be an inhibitory domain that sterically blocks the binding of FGF ligands to the binding pocket located between the D2 and D3 domains [71]. We hypothesize that the FGFR1 mutation has decreased ability to open the binding pocket for FGF21; however, future studies are needed to determine the direct effect of these mutations on FGF21 binding. The KLB mutation maps to a highly conserved region of the signal peptide portion of the protein. Interestingly, KLB hosts an exceptionally long signal peptide (52 amino acids) [72]. Literature suggests that signal peptides of this length may indicate an important role in protein processing or localization [73]. Perhaps the KLB mutation interferes with cleavage of the signal peptide or localization of the protein to the cell surface. Further studies are warranted to evaluate these possibilities.

In this family, we propose a digenic mechanism of inheritance, in which the FGFR1 and KLB mutations are acting synergistically. Genetic diseases may result from monogenic (single gene) and polygenic (multiple genes) variation. Typically, polygenic conditions are less severe than monogenic disorders [74]. Digenic inheritance (also known as synergistic heterozygosity) proposes that multiple partial defects have an additive effect, resulting in an abnormal phenotype [74, 75]. A digenically inherited insulin resistance syndrome resulting from heterozygous variants in the *PPARG* and *PPP1R3A* genes has been described previously [76].

In large databases of adults, the frequencies of these variants are relatively rare. According to gnomAD (http://gnomad.broadinstitute.org), the minor allele frequency (MAF) of the *FGFR1* p.V102I variant is 0.00039. However, this variant is enriched in the Latino subset with a MAF of 0.00045. The MAF of the *KLB* p.S9Y variant follows a similar pattern (0.002 of all individuals in gnomAD with increased frequency among the African subset MAF = 0.02), with 7 individuals homozygous for this allele reported.

It is unclear what is the best treatment strategy to pursue for individuals with IMPA. In our proband’s case, we have sought to use metformin to improve insulin sensitivity. Oral contraceptive pills and spironolactone were successfully used to stimulate regular menstrual cycles and reduce hyperandrogenism. One intriguing possibility is to use a thiazolidinedione (ie, pioglitazone), as it is a potent inducer of hepatic FGF21 [77]. Additionally, several FGF21 analogs are currently being studied in both phase 1 and 2 clinical trials [78]. Potentially, such compounds could be used under compassionate use in individuals with IMPA. However, we think that it is unlikely that increasing FGF21 will be able to overcome the defect in the FGFR1-KLB receptor complex.

In summary, we have characterized a patient with a rare insulin resistance and overgrowth syndrome (insulin-mediated pseudoacromegaly). We demonstrate that the affected proband carries digenic loss of function mutations in FGFR1 and KLB potentially causing endocrine specific FGF21 resistance. This is the first report demonstrating a potential genetic and molecular etiology for this rare insulin resistance syndrome.

## Data Availability

Restrictions apply to the availability of data generated or analyzed during this study to preserve patient confidentiality. The corresponding author will on request detail the restrictions and any conditions under which access to some data may be provided.

## Abbreviations

BMI: body mass index
CNS: central nervous system
ELISA: enzyme-linked immunosorbent assay
FGF21: fibroblast growth factor 21
FGFR1: fibroblast growth factor receptor 1
GFP: green fluorescent protein
IGF1: insulin-like growth factor 1
IGFBP3: insulin-like growth factor binding protein 3
IMPA: insulin-mediated pseudoacromegaly
INSR: insulin receptor
KLB: beta-Klotho
MAF: minor allele frequency
pERK: phosphorylated extracellular signal-regulated kinase (ERK)

## Additional Information

***Disclosure Summary:*** The authors have nothing to disclose.
